# Fast-Track Systems Improve Timely Carotid Endarterectomy in Stroke Prevention Outpatients

**DOI:** 10.1017/cjn.2016.270

**Published:** 2016-07-26

**Authors:** Sophia Gocan, Aline Bourgoin, Dylan Blacquiere, Rany Shamloul, Dar Dowlatshahi, Grant Stotts

**Affiliations:** 1 The Ottawa Hospital, Champlain Regional Stroke Network, Ottawa Hospital Research Institute, Ottawa, Ontario, Canada; 2 Saint John Regional Hospital, Horizon Health Network, Division of Neurology, Saint John, New Brunswick, Canada; 3 Ottawa Stroke Research Group, Ottawa Hospital Research Institute, Ottawa, Ontario, Canada; 4 The Ottawa Hospital, Division of Neurology, Ottawa Hospital Research Institute, Ottawa, Ontario, Canada.

**Keywords:** Cerebrovascular disease, Carotid endarterectomy, Stroke prevention

## Abstract

***Background:*** For optimal stroke prevention, best practices guidelines recommend carotid endarterectomy (CEA) for symptomatic patients within two weeks; however, 2013 Ontario data indicated that only 9% of eligible patients from outpatient Stroke Prevention Clinics (SPCs) achieved this target. The goal of our study was to identify modifiable system factors that could enhance the quality and timeliness of care among patients needing urgent CEA. ***Methods:*** We conducted a retrospective chart review of transient ischemic attack/stroke patients assessed in Champlain Local Health Integrated Network SPCs between 2011 and 2014 who subsequently underwent CEA. Descriptive statistics were used to define patient characteristics, timelines from symptom onset to CEA, and system factors that contributed to delays or improvements in care. Multivariate analysis was used to determine statistically significant variations between groups. ***Results:*** Seventy-five records were eligible for study inclusion. Median time from initial symptoms to CEA was 31 days, with 21.3% of patients undergoing surgery within 2 weeks. Significant delays were common in patient presentation and assessment following symptom onset, wait times for vascular imaging and neurological assessment, and time from surgical assessment to CEA completion. Rapid testing and triage, coupled with collaborative initiatives among SPC, surgical, and radiology teams were associated with significantly improved timelines. ***Conclusions:*** Success factors for rapid CEA are multifaceted, including system changes that address public awareness of stroke and 911 response, improvements in vascular imaging access, and redesign of clinical services to promote collaboration and fast-tracking of care. Implementation of performance measures to monitor and guide clinical innovations is recommended.

## RÉSUMÉ

**Prévention des accidents vasculaires cérébraux chez des patients externes au moyen de l’endartériectomie carotidienne: des innovations systémiques peuvent améliorer la rapidité de leur prise en charge.** ***Contexte:*** Pour prévenir de façon adéquate les accidents vasculaires cérébraux (AVC), les lignes directrices sur les pratiques exemplaires recommandent l’endartériectomie carotidienne (EAC) pour les patients symptomatiques, et ce, dans un délai de deux semaines. Cela dit, des données ontariennes de 2013 indiquent que seulement 9% des patients admissibles aux services externes des cliniques de prévention des AVC étaient visés par cet objectif. Le but de notre étude a donc été de déterminer les facteurs systémiques dont les modifications seraient susceptibles d’améliorer la qualité et la rapidité des soins donnés aux patients qui nécessitent de manière urgente une EAC. ***Méthodes:*** Nous avons procédé à une analyse rétrospective des dossiers de patients victimes d’une ischémie cérébrale transitoire (ICT) ou d’un AVC. Ces patients avaient été évalués entre 2011 et 2014 dans des cliniques de prévention des AVC du Réseau local d’intégration des services de santé (RLISS) de Champlain et ont ultérieurement subi une EAC. Nous avons ainsi utilisé des statistiques descriptives pour définir les caractéristiques des divers patients, les délais entre l’apparition des premiers symptômes et l’exécution d’une EAC et les facteurs systémiques ayant contribué à ces délais ou à une prise en charge accélérée des patients. Nous avons également utilisé l’analyse multi-variable pour relever les variations statistiquement significatives entre les groupes. ***Résultats:*** Soixante-quinze dossiers ont été pris en compte dans cette étude. La moyenne des délais entre l’apparition des premiers symptômes et une EAC était de 31 jours, 21,3% des patients subissant une chirurgie dans un délai de deux semaines. Des délais importants étaient fréquents en ce qui concerne la prise en charge et l’évaluation des patients à la suite de l’apparition des premiers symptômes. Il en va de même avec la possibilité de bénéficier de tests d’imagerie vasculaire et d’une évaluation neurologique ainsi que d’une évaluation en vue de l’exécution d’une EAC. À cet égard, des interventions de dépistage et de triage plus rapides jumelées à des initiatives de collaboration entre les équipes des cliniques de prévention des AVC et celles œuvrant dans les services de chirurgie et de radiologie ont été associées à des délais d’intervention sensiblement améliorés. ***Conclusions:*** Les facteurs qui conduisent à une prompte EAC sont variés. Il faut notamment mentionner des changements d’ordre systémique tenant compte de la sensibilisation du public par rapport aux AVC et de la rapidité du service d’urgence 9-1-1 mais aussi de l’amélioration de l’accès aux test d’imagerie vasculaire et d’une restructuration des services cliniques afin de promouvoir la collaboration entre professionnels et une prise en charge accélérée des patients. Voilà pourquoi il est recommandé de mettre en œuvre des mesures du rendement afin d’orienter et de suivre ces innovations de type clinique.

Delays in the delivery of urgent carotid endarterectomy (CEA) for secondary stroke prevention are well-documented.^1^ ^-^ ^4^ This is highly significant for patients presenting with transient ischemic attack (TIA) or stroke who demonstrate moderate to severe ipsilateral, symptomatic carotid artery stenosis because their 2-day risk of stroke may be as high as 5.2%, 14-day risk may be as high as 11%,^5^ and the 90-day risk of stroke ranges between 20% and 30%.^6^ ^,^ ^7^ Although CEA has been shown to significantly reduce stroke risk, its effectiveness is highly time-dependent, with a number needed to treat of five among those who undergo surgery within 2 weeks, compared with a number needed to treat of 125 among those receiving surgery after more than 12 weeks.^8^

For optimal stroke prevention, international best practice guidelines recommend CEA intervention as soon as safe and possible for appropriate candidates, with a target of less than 2 weeks.^9^ ^-^ ^11^ However, very few health centers consistently achieve this benchmark, particularly in the outpatient setting. The 2013 Ontario Stroke Evaluation Report included data from more than 16,000 patients seen at 40 outpatient Stroke Prevention Clinic (SPC) sites between 2011 and 2012. Only 9% of patients seen within these centres received their CEA within 2 weeks; the median wait time to CEA was 50 days.^4^

We conducted this study to identify modifiable system factors and clinical processes that could contribute to enhancements in the quality and timeliness of care among SPC patients in need of urgent CEA surgery. We hypothesized that a review of health records, including a critical analysis of timelines from symptoms onset to CEA, would identify clinically relevant system factors and processes amenable to change to improve the achievement of established benchmarks.

## Methods

### Data Collection

We retrospectively reviewed the health records of patients referred to or assessed at four Champlain outpatient SPC sites following TIA or minor stroke between fiscal years (FY) 2011-2012 to 2013-2014 who subsequently underwent CEA at The Ottawa Hospital (TOH). TOH is a Canadian, multisite, academic health sciences centre that serves 1.2 million people across the Champlain Local Health Integration Network (LHIN) in Eastern Ontario. It is the only centre that offers CEA in the Champlain region.

Patients were identified using administrative data through the Canadian Stroke Network Stroke Performance Indicators for Reporting, Improvement and Translation portal. This database included information on all Champlain SPC patients, including those who received a carotid intervention from FY 2011-2012 to 2012-2013. For FY 2013-2014, patients were identified through a retrospective review of each TOH SPC chart. Patients were excluded if they were <19 years of age, directly admitted to the hospital from the emergency department for evaluation or after completed stroke, had a stroke/TIA during an inpatient stay, or were identified as having asymptomatic carotid stenosis by the stroke physician.

**Figure 1:**
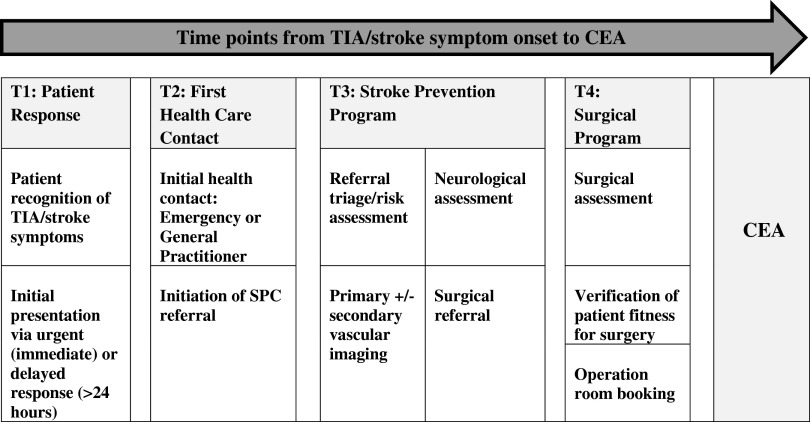
Patient time points, system milestones, and key activities from TIA/stroke symptom onset to CEA. *T=time point. Acronyms: T (Time point), TIA (Transient ischemic attack), SPC (Stroke Prevention Clinic), CEA (Carotid Endarterectomy).

We used a standardized case report form to extract patient characteristics, details around vascular imaging, triage levels, surgical variables, characteristics of the presenting event, and details regarding adverse events. We also abstracted dates for the following time points: (1) initial symptom onset, (2) most recent symptoms, (3) initial patient presentation, (4) stroke physician/SPC referral, (5) stroke physician/SPC assessment, (6) initially scheduled and actual SPC appointment, (7) primary vascular imaging, (8) secondary vascular imaging, (9) surgical referral, (10) surgical assessment, and (11) CEA. Figure 1 depicts typical time points, system milestones, and key activities of patient flow within Champlain SPCs from time of symptom onset to CEA.

Primary study outcomes included timelines for patients to reach defined time points as described previously. In addition, this included a review of the clinical and system factors influencing either delayed or expedited care such as triage category, recurrent vascular events, SPC referral source, CEA operation priority code, and team collaboration factors. Secondary outcomes included the number of adverse events (recurrent stroke, coronary/vascular complication, death, hospitalization) between initial TIA/stroke event and CEA procedure.

### Statistical Analysis

Descriptive results are expressed as frequencies (percentages) and median values (± ranges) where appropriate. In addition, we used the Mann Whitney *U* test or Kruskal-Wallis test to determine statistically significant differences between groups. Spearman’s rank correlation test was used to determine the relationship between timelines to CEA and patient age. All analyses were performed using SPSS, version 20 (Statistical Package for the Social Sciences, Chicago), with a p value ≤0.05 deemed significant.

## Results

Between FY 2011-2012 and 2013-2014, a total of 5136 patients were seen at Champlain LHIN SPCs. From this group, 75 patients met inclusion criteria for our study (1.5%). Patient demographics and medical history details are described in Table 1. Table 2 outlines patient characteristics and reports the median time in days, with interquartile range (IQR), for each group to progress from symptom onset to CEA as well as the statistical significance of these values.

**Table 1:** Patient demographics and medical history

Demographics	Median (SD)
Age	71 (8.46)
**Medical history**	**Number (%)**
Hypertension	59 (78.7)
Dyslipidemia	52 (69.3)
Diabetes mellitus	23 (30.7)
Current smoker (within 6 months)	21 (28)
Excess alcohol use (above Canada’s low-risk guidelines)	11 (14.7)
Contralateral carotid stenosis >50%	39 (52)
Coronary artery disease	22 (29.3)
Peripheral vascular disease	18 (24)
Atrial fibrillation	5 (6.7)
Prior TIA/stroke	11 (14.7)
Prior carotid endarterectomy	4 (5.3)

SD=standard deviation.

**Table 2:** Patient characteristics and timelines from initial symptoms to CEA

Patient characteristics	Number (%)	Median time to CEA (days)	p value
Male	46 (61.3)	25.5 (14-44.8)	0.087
Female	29 (38.7)	34 (22-57.5)	
Rural postal code	19 (25.3)	31 (14-42)	0.966
Urban postal code	56 (74.7)	28.5 (16.5-53)	
**Distribution of patients by stroke prevention clinic site**	**Number (%)**	**Median days to CEA (IQR)**	**p value**
The Ottawa Hospital	69 (92)	27 (15-52)	0.802
Queensway Carleton Hospital	4 (5.3)	35.5 (25.8-50.5)	
Pembroke Regional Hospital	2 (2.7)	32.5 (31)	
Hawkesbury General Hospital	0 (0)	N/A	
**Diagnosis according to stroke physician**			
Stroke	17 (22.7)	42 (21.5-69)	0.208
Hemispheric TIA	44 (58.7)	31 (14-44.8)	
Retinal event only	14 (18.7)	22 (15.8-44.3)	
**Duration of index event**			
0-59 minutes	44 (58.7)	34 (19.3-57)	0.145
>60 minutes	31 (41.3)	27 (11-43)	
**Cerebrovascular event pattern**			
Recurrent or crescendo TIA	26 (34.7)	23 (13.5-40)	0.161
Single TIA/stroke event	49 (65.3)	34 (19-55.5)	
**Degree of symptomatic stenosis**			
50-70%	14 (18.7)	33 (17.3-46.3)	0.814
>70%	61 (81.3)	30.5 (16.8-54)	

Statistical significance was calculated using the Spearman rank test (for age) and Mann Whitney *U* or Kruskal-Wallis for categorical/nominal data.

### Timelines to CEA

**Figure 2:**
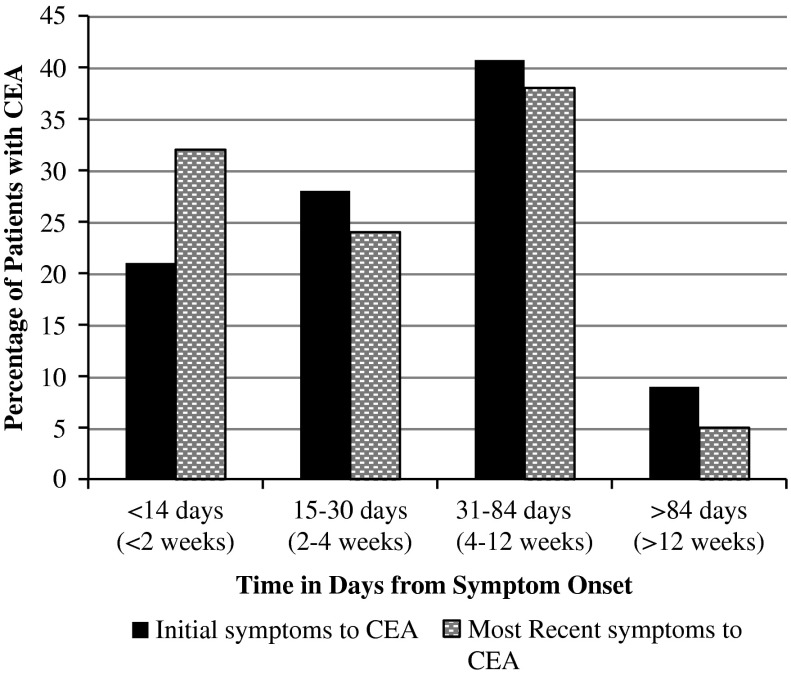
Percentage of patients with completed CEA (in days) from symptom onset. CEA=Carotid Endarterectomy

The median time to CEA was 31 days (IQR, 18-58 days) from initial symptom onset of TIA/stroke symptoms, and 25 days (IQR, 12-54 days) from time of most recent symptoms. CEA treatment within 2 weeks was achieved for 21% and 32% of patients when measured from time of initial symptoms and most recent symptoms, respectively (Figure 2). Recurrent TIA events (defined as two or more events within 2 weeks) and crescendo TIA events (defined as two or more events within 24 hours) were relatively common in our study cohort and occurred in 26 patients (36%). Half of these TIA events occurred before patient presentation and/or SPC referral. There were no statistically significant differences seen in time to CEA among those who experienced recurrent events compared with patients who experienced a single TIA/stroke event.

### Timelines for Patients to Reach Defined Time Points

**Figure 3:**
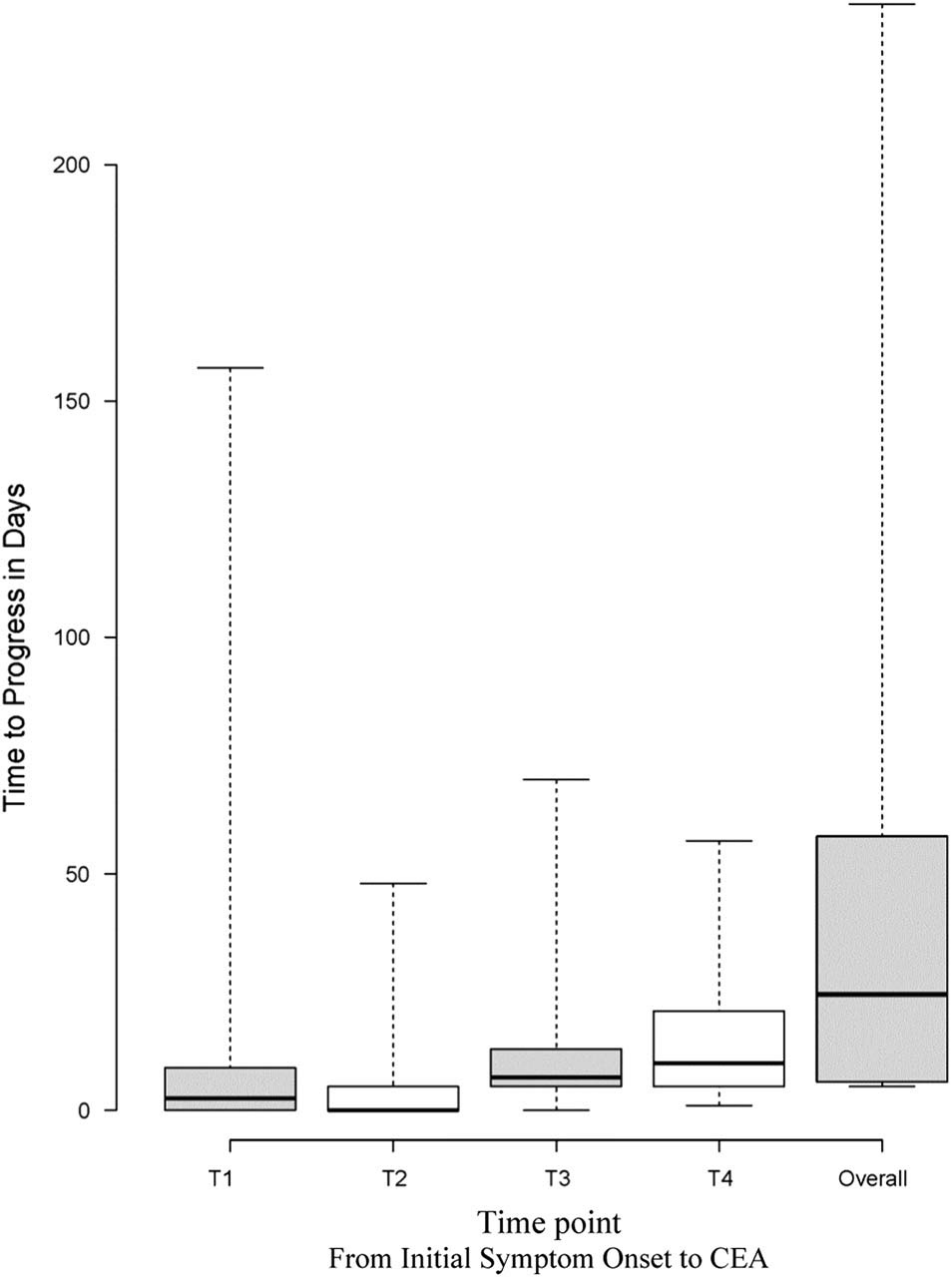
Time to progress within specified time points. T=Time point. Theses time points were defined as follows: T1 - Patient recognition of symptoms and initial presentation; T2: Emergency or General Practioner assessment and SPC referral; T3: SPC/stroke physician assessment and surgical referral; T4: Time from surgical referral to CEA and Overall: Time from initial symptom onset to CEA. The upper and lower whiskers represent the range in days. The upper box, lower box and middle line represent the 3rd quartile, 1st quartile and median respectively.

The time it took for patients to progress through defined time points is presented as a box plot in Figure 3. We examined how many days it took to progress through each of the following periods: T1: patient recognition of symptoms and initial presentation; T2: emergency or general practitioner assessment and SPC referral; T3: SPC/stroke physician assessment and surgical referral; T4: time from surgical referral to CEA; and overall: time from initial symptoms to CEA.

The longest time points between symptom onset and CEA included the period from surgical referral to CEA (T4: mean, 14 days; median, 14 days; IQR 6-21), the period from SPC referral to surgical referral (T3: mean, 10.9 days; median, 8 days; IQR 6-13), and the period from patient recognition of symptoms to initial presentation (T1: mean, 8.97 days; median, 0 days; IQR 0-9), followed by the period from initial assessment to SPC referral (T2: mean, 3.19 days; median, 0 days; IQR 0-0).

Within T3, the time from SPC referral to primary vascular imaging was prolonged (mean, 7.88 days; median, 6 days; IQR 3-11). Within T4, the time from surgical referral to surgical assessment was relatively short (mean, 3.58 days; median, 2 days; IQR 0-7) when compared with the time from surgical assessment to CEA (mean, 11.72 days; median, 9 days; IQR 3-15). Although T1 had a median of 0 days from initial onset of symptoms to initial health care presentation, almost half of the patient cohort (44%) did not present on the day of their TIA/stroke event. Furthermore, more than half of those who delayed initial presentation, waited >72 hours for their initial health care encounter.

### Additional Clinical Factors Affecting Timelines to CEA

Additional clinical factors affecting timelines to CEA are described in this section, with significance values defined in Table 3.

**Table 3:** Clinical factors affecting time from symptoms to CEA

Clinical factor	Number (%)	Median days to CEA (IQR)	p value
**Patient site of initial presentation/SPC referral source**			
Emergency department	40 (53.3)	24 (14.3-34.8)	0.017*
Primary care	24 (32.0)	43.5 (19.3-70.8)	
Specialist	11 (14.7)	40 (20-70)	
**SPC triage category**			
Low risk	4 (5.3)	30 (16.3-55.8)	0.990
Moderate risk	13 (17.3)	34 (14.5-49)	
High risk	52 (69.3)	24 (14.5-54)	
**Collaboration between SPC and carotid surgery teams**			
Same-day patient assessment by SPC and surgery teams	16 (21.3)	17.5 (8-33)	0.012*
Patient assessment by SPC and surgery teams on different days	59 (78.7)	34 (20-54)	
**Collaboration between carotid surgeons**			
Different surgeon for assessment and surgery (“single queue”)	19 (25.3)	19 (11-24)	0.001*
Same surgeon for assessment and surgery	56 (74.7)	38.5 (20-57.8)	
**Operating priority code**			
Elective	50 (66.7)	40 (24-58.3)	0.001*
Urgent	17 (22.7)	14 (8-22.5)	
Work in as outpatient	8 (10.6)	22.5 (15.3-55.5)	

Statistical significance was calculated using the Mann Whitney *U* or Kruskal-Wallis test. *Indicates a significant p value of ≤0.05.

Patients who presented to the emergency department following their index TIA/stroke event progressed more quickly from initial symptom onset to both SPC referral and CEA when compared with those presenting either to their general practitioner or specialist. On average, patients who presented to the emergency department (53%) following their initial TIA/stroke symptoms had their first health care contact significantly earlier (assessed initially at 1 day) compared with those who presented initially to their general practitioner or specialist (assessed initially at 17 and 21 days, respectively).

Patient triage levels in the SPC fit into three categories of urgency: low, moderate, or high. Within this study, 52 patients were triaged high, 13 triaged moderate, and 4 were triaged low. Patients who were triaged high were seen in the SPC 6 to 10 days sooner than those triaged moderate or low, although these differences were not found to be statistically significant. In addition, 25.3% of patients (n=19) had their SPC visit advanced from their originally scheduled date. In the majority of these cases (75%), visits were retriaged and rescheduled by SPC staff urgently following the patients’ vascular imaging study.

Patients who were assessed in the SPC by the stroke physician, and subsequently by the carotid surgeon on the same date, had their CEA more quickly (17.5 days) compared with those who had their assessments on different dates (34 days).

Surgical collaboration involved the use of a “single-queue” model. Rather than remaining under the care of a single surgeon who may have an extended wait time, selected patients were scheduled on a priority basis with the surgical team member who had the next available operation room space. This single-queue approach was more expedient than keeping the patient with the original consulting surgeon (19 days compared with 38.5 days).

Patients were assigned one of three operating priority codes: urgent, elective, or work in as outpatient. Those assigned an urgent code rather than an elective or work in as outpatient code had a significantly reduced time from symptom onset to CEA and were more likely to meet the targets set out by stroke best practice guidelines (14 days to CEA rather than 40 or 22.5 days, respectively).

### Secondary Outcomes

Seven patients (9.3%) were admitted for medical management or stabilization of carotid disease. Five of these admissions occurred directly from the stroke prevention clinic, and two were related to patients who presented to the emergency department with recurrent TIA symptoms. Table 4 highlights the incidence of adverse events in our study cohort. There were no coronary/vascular complications or deaths.

**Table 4:** Adverse events between SPC referral and CEA

Adverse events	N (%)
Stroke events	0 (0)
Transient ischemic attack events	13 (17.3)

#### Interpretation

The proportion of eligible patients for our study included 1.5% of the overall SPC cohort. This percentage is consistent with data published in the Ontario Stroke Evaluation Report 2013, which indicated that among patients seen at Ontario Stroke Prevention Clinics, 235 of 15,534 patients (1.5%) received CEA following their SPC visit.^4^

Within this study cohort, only 21% of patients received their CEA within 2 weeks of their initial symptoms, meeting the best practice target that was in place at the time of our research study. Even among patients with recurrent TIA symptoms, significant gains were not achieved, with only 32% reaching this target. The most recently published Canadian best practice recommendations for stroke, recognizing the critically short window in which to prevent recurrent stroke events, have tightened these timelines further and indicated that patients with mild stroke or TIA should have CEA performed within 48 hours of symptom onset.^11^ To achieve a 48-hour target, several barriers need to be addressed throughout the systems of care influencing patient progress to urgent CEA, beginning with a strong public awareness of stroke.

Many patients in our study neglected to respond urgently to their TIA/stroke symptoms, which contributed substantially to delays in care. Only 56% of patients presented to a health professional on the day of their initial event and only 53% of patients went to the emergency department for their first health care contact. Our study complements prior research that has identified patient delays in seeking medical attention as one of the most common causes of extended timelines to CEA.^12^ ^,^ ^13^ In line with previous studies, our data also suggest that location of patient presentation is important, with referrals from the emergency department predicting significantly shorter wait times to CEA.^1^ ^,^ ^14^ Delays to first health assessment contribute to delays in the completion of urgent diagnostic testing, identification of stroke etiology, and the initiation of preventive medical and surgical treatments that reduce stroke recurrence. These results emphasize the importance of public awareness regarding stroke symptoms and an urgent/911 response as the foundation of “fast-track” care.

After a patient has been referred to the SPC for TIA/stroke workup and assessment, other “fast-track” strategies were associated with shorter timelines to CEA. In particular, rapid vascular imaging and collaboration between health teams such as radiology, SPC, and carotid surgery teams were associated with significantly shorter timelines to CEA. For example, 20% of patients in our study had their SPC visit advanced once critical vascular imaging results were communicated by radiology. Unfortunately, this imaging took place on average 6 days after the initial event; therefore, earlier access to vascular imaging stands out as a target for clinical improvement. Canadian best practice recommendations identify the importance of vascular imaging as a critical component of the initial patient assessment.^11^ ^,^ ^15^ In this same thread, Canadian stroke clinicians and leaders are calling for paradigm shifts in care and the reorganization of stroke systems so that the etiology of stroke and the corresponding treatments can be identified, with preventive treatments implemented within the first day of TIA/stroke symptom onset.^16^

In our study, same-day assessment by stroke physician and carotid surgeons also resulted in average time reductions of 16.5 days to CEA. Previous research in which service reconfiguration included “fast-tracked” patient vascular imaging, SPC access, and admission to surgery directly from the SPC resulted in substantial reductions in wait times with 83% of the study cohort getting CEA within 2 weeks.^17^ ^,^ ^18^

Surgical collaboration that involved different surgeons for assessment and operating room encounters, using a “single-queue” model, demonstrated shorter CEA timelines by an average of 19.5 days. This practice has previously demonstrated success.^18^ Single-queue booking for surgery, with a focus on urgent surgical access, may represent one of the multifaceted clinical strategies that can lead to reduced CEA timelines and stroke recurrence rates. Its use may benefit from further research and replication in other institutions. In addition, an urgent operative code corresponded with patient timelines to CEA, which were considerably shorter than those assigned a work in as outpatient or elective status. Given the imminent danger of recurrent stroke in this high-risk population of patients, hospital policies and protocols should be established to classify carotid endarterectomy (for severe symptomatic stenosis) as an emergent procedure, with priority allocation of operation room time.

Several authors have suggested performance measurement as a key tool in making simple, but effective changes to shorten the delay from symptom onset to surgery.^19^ ^,^ ^20^ In particular, taking a “real-time,” proactive approach to systematically track and modify clinically processes from symptom onset to CEA, rather than relying on a retrospective review of care. This has been referred to as symptom to knife time in the literature, and follows a similar approach as the quality improvement measures that have been adopted to improve door to needle times for thrombolysis delivery in acute stroke.^19^ ^,^ ^20^

#### Limitations of the Study

The retrospective nature of our study is one important limitation to consider. In addition, the sample size is relatively small, thereby reducing the power to detect significant associations. Another consideration is that our region may use unique patient flow processes and clinical care structures that contributed to CEA timeline efficiencies or delays. Patients who were admitted directly from the emergency department were excluded from our study to ensure the factors examined were representative of the outpatient flow process. In excluding inpatient cases, our study may have captured a lower risk group of patients biased towards longer median CEA wait times. However, one strength within our cohort is that it included patients from three SPC sites within the Champlain LHIN who were enrolled in a consecutive basis, thereby reducing the risk of bias. Further research involving other outpatient clinics across Ontario and internationally would assist in verification of results and identification of additional factors to improve benchmark targets.

## Conclusion And Future Directions In The Area Of Study

CEA remains the most effective method of stroke prevention for patients with symptomatic moderate- to high-grade carotid stenosis. All efforts to speed up patients’ stroke prevention care from symptom onset to CEA are of great importance to minimize the chances of further stroke events. The factors that will contribute to greater success are multifaceted and include system changes that address public awareness of stroke and 911 response, improvements in immediate access to vascular imaging, redesign of clinical services to allow for greater collaboration and fast-tracking of care, and implementation of performance measures to track and improve symptom to knife time. Together, these changes have the potential to improve patient safety, quality of care, and most important, clinical outcomes. Further research in this area is needed to improve outcomes at individual centres and to replicate successes at provincial and national levels.

## References

[1] Jetty P, Husereau D, Kubelik D, et al Wait times among patients with symptomatic carotid artery stenosis requiring carotid endarterectomy for stroke prevention. J Vasc Surg. 2012;56:661-667. 22608182 10.1016/j.jvs.2012.03.001

[2] Gladstone DJ, Oh J, Fang J, et al Urgency of carotid endarterectomy for secondary stroke prevention: results from the Registry of the Canadian Stroke Network. Stroke. 2009;40:2776-2782. 19542057 10.1161/STROKEAHA.109.547497

[3] Dyer E, Lownie S, Ferguson G. Wait times for carotid endarterectomy, London Ontario 2006-2007. Can J Neurol Sci. 2013;40:330-333. 23603167 10.1017/s0317167100014268

[4] Hall R, Khan F, O’Callaghan C, et al. Ontario Stroke Evaluation Report 2013: spotlight on secondary stroke prevention and care. 1-274. 2015. Toronto, Ontario, Institute for Clinical Evaluative Sciences.

[5] Johansson EP, Arnerlov C, Wester P. Risk of recurrent stroke before carotid endarterectomy: the ANSYSCAP study. Int J Stroke. 2013;8:220-227. 22494778 10.1111/j.1747-4949.2012.00790.x

[6] Eliasziw M, Kennedy J, Hill MD, et al Early risk of stroke after a transient ischemic attack in patients with internal carotid artery disease. CMAJ. 2004;170:1105-1109. 15051694 10.1503/cmaj.1030460 PMC374217

[7] Fairhead JF, Mehta Z, Rothwell PM. Population-based study of delays in carotid imaging and surgery and the risk of recurrent stroke. Neurology. 2005;65:371-375. 16087900 10.1212/01.wnl.0000170368.82460.b4

[8] Rothwell PM, Eliasziw M, Gutnikov SA, et al Endarterectomy for symptomatic carotid stenosis in relation to clinical subgroups and timing of surgery. Lancet. 2004;363:915-924. 15043958 10.1016/S0140-6736(04)15785-1

[9] Kernan WN, Ovbiagele B, Black HR, et al Guidelines for the prevention of stroke in patients with stroke and transient ischemic attack: a guideline for healthcare professionals from the American Heart Association/American Stroke Association. Stroke. 2014;45:2160-2236. 24788967 10.1161/STR.0000000000000024

[10] Scottish Intercollegiate Guidelines Network. Management of patients with stroke or TIA: assessment, investigation, immediate management and secondary prevention. A national clinical guideline. 1-108. 2015. Edinburgh, Scotland.

[11] Coutts SB, Wein TH, Lindsay MP, et al Canadian Stroke Best Practice Recommendations: secondary prevention of stroke guidelines, update 2014. Int J Stroke. 2015;10:282-291. 25535808 10.1111/ijs.12439

[12] Gaba KA, Syed MJ, Raza Z. Reducing the delay for carotid endarterectomy in South-East Scotland. Surgeon. 2014;12:11-16. 24262715 10.1016/j.surge.2013.09.008

[13] Khashram M, Roake JA, Lewis DR. Patient flow to carotid endarterectomy: hastening the patient journey. ANZ J Surg. 2010;80:406-410. 20618192 10.1111/j.1445-2197.2010.05308.x

[14] Blacquiere D, Sharma M, Jetty P. Delays in carotid endarterectomy: the process is the problem. Can J Neurol Sci. 2013;40:585-589. 23786745 10.1017/s0317167100014712

[15] Casaubon LK, Boulanger JM, Blacquiere D, et al Canadian Stroke Best Practice Recommendations: Hyperacute Stroke Care Guidelines, Update 2015. Int J Stroke. 2015;10:924-940. 26148019 10.1111/ijs.12551

[16] Kamal N, Hill MD, Blacquiere DP, et al Rapid assessment and treatment of transient ischemic attacks and minor stroke in Canadian emergency departments: time for a paradigm shift. Stroke. 2015;46:2987-2990. 26316346 10.1161/STROKEAHA.115.010454

[17] Ali M, Stephenson J, Naylor AR. Delay prior to expedited carotid endarterectomy: a prospective audit of practice. Eur J Vasc Endovasc Surg. 2013;46:404-410. 23973275 10.1016/j.ejvs.2013.07.015

[18] Abbas K, Vohra RS, Salhab M, et al A strategy to meet the ‘two-week’ target for carotid endarterectomy in symptomatic patients. Clin Med. 2011;11:452-455. 10.7861/clinmedicine.11-5-452 PMC4954238 22034704

[19] Noronen K, Vikatmaa P, Sairanen T, et al Decreasing the delay to carotid endarterectomy in symptomatic patients with carotid stenosis--outcome of an intervention. Eur J Vasc Endovasc Surg. 2012;44:261-266. 22841357 10.1016/j.ejvs.2012.06.014

[20] Vikatmaa P, Sairanen T, Lindholm JM, et al Structure of delay in carotid surgery—an observational study. Eur J Vasc Endovasc Surg. 2011;42:273-279. 21620740 10.1016/j.ejvs.2011.04.021

